# Evidence of *Trem2* Variant Associated with Triple Risk of Alzheimer’s Disease

**DOI:** 10.1371/journal.pone.0092648

**Published:** 2014-03-24

**Authors:** Zainularifeen Abduljaleel, Faisal A. Al-Allaf, Wajahatullah Khan, Mohammad Athar, Naiyer Shahzad, Mohiuddin M. Taher, Mohamed Elrobh, Mohammed S. Alanazi, Waseem El-Huneidi

**Affiliations:** 1 Department of Medical Genetics, Faculty of Medicine, Umm Al-Qura University, Makkah, Saudi Arabia; 2 Department of Basic Sciences, College of Science and Health Professions, King Saud Bin Abdulaziz University for Health Sciences, Riyadh, Saudi Arabia; 3 Department of Pharmacology and Toxicology, Faculty of Medicine, Umm Al-Qura University, Makkah, Saudi Arabia; 4 Genome Research Chair Unit, Department of Biochemistry, College of Science, King Saud University, Riyadh, Saudi Arabia; Massachusetts General Hospital and Harvard Medical School, United States of America

## Abstract

Alzheimer’s disease is one of the main causes of dementia among elderly individuals and leads to the neurodegeneration of different areas of the brain, resulting in memory impairments and loss of cognitive functions. Recently, a rare variant that is associated with 3-fold higher risk of Alzheimer’s disease onset has been found. The rare variant discovered is a missense mutation in the loop region of exon 2 of *Trem2* (rs75932628-T, Arg47His). The aim of this study was to investigate the evidence for potential structural and functional significance of *Trem2* gene variant (Arg47His) through molecular dynamics simulations. Our results showed the alteration caused due to the variant in TREM2 protein has significant effect on the ligand binding affinity as well as structural configuration. Based on molecular dynamics (MD) simulation under salvation, the results confirmed that native form of the variant (Arg47His) might be responsible for improved compactness, hence thereby improved protein folding. Protein simulation was carried out at different temperatures. At 300K, the deviation of the theoretical model of TREM2 protein increased from 2.0 Å at 10 ns. In contrast, the deviation of the Arg47His mutation was maintained at 1.2 Å until the end of the simulation (t = 10 ns), which indicated that Arg47His had reached its folded state. The mutant residue was a highly conserved region and was similar to “immunoglobulin V-set” and “immunoglobulin-like folds”. Taken together, the result from this study provides a biophysical insight on how the studied variant could contribute to the genetic susceptibility to Alzheimer’s disease.

## Introduction

Almost 30 million people live with Alzheimer’s disease worldwide, causing staggering healthcare burden, which is expected to quadruple by 2050. Alzheimer’s disease is a challenging brain damaging disease, gradually affecting person’s memory and ability to spend life independently. There is no effective treatment, and scientists are still trying to determine the underlying mechanism of the disease. Recently published research has provided some hope, by showing that a genetic mutation confers a significantly higher risk of Alzheimer’s disease, whereas others may possibly be prevented from developing this condition [1], [2]. The disease results in the accumulation of protein tangles and plaques in and out of brain cells, affecting neurons signaling process. Two recently published reports [1], [2] show an association between Alzheimer’s disease and a rare gene mutation that disrupts the immune system's inflammatory mechanism. The new finding provides further evidence regarding the emerging theory that indicates a possible involvement of the immune system in the Alzheimer’s disease progression. The *Trem2* gene responsible for triggering receptor expressed on monocytes 2 and certain loss of function mutations has been shown to be link with an autosomal recessive condition of premature dementia. Although TREM2 is expressed throughout the central nervous system but specifically expressed in the white matter. Its protein has similarities with immunoglobulin and performs as a phagocytic receptor for bacteria as well as a regulator of the inflammatory stimulus. The protein plays an essential role in removing the neural debris on microglia [3]. Recently, Guerreiro et al. (2013) identified a causative *Trem2* mutant allele in a study comprised of 1,092 patients with Alzheimer’s disease and 1,107 controls through Meta analyses. These authors examined unrelated databases of patients and controls to replicate and verify their initial findings. They further analyzed the differential gene expression of *Trem2* over wide range of human brain areas in mice with Alzheimer’s disease and compared it with the control mice to study the genetic mutation and disease mechanism. They concluded that *Trem2* rare heterozygous variants were linked with a significantly elevated risk of Alzheimer’s disease [2]. In another study by Jonsson et al. (2013), in which they analyzed 2,261 Icelanders and concluded that a rare variant (rs75932628; Arg47His) in *Trem2* showed 3-fold higher risk of Alzheimer’s disease over the control. They observed similar trend when they studied a combined group of 2,000 individuals from four diverse groups in the U.S. and Europe. Their finding strongly implicated the variant in disease pathogenesis probably as a result of impaired containment of inflammatory mechanism.

In the present study, we examined *in silico* structural and functional studies using molecular dynamics (MD) simulation for the variant rs75932628. We further compared the native protein structure and mutant structures and their solvent accessibility including secondary structures. Our *in silico* examination suggested the presence of further deleterious mutations in intergenic genes, which may possibly have an impact on the structure and function of proteins with known roles in Alzheimer’s disease.

## Materials and Methods

### Dataset Sources

We utilized the results of the dataset from two recently published reports comprised of both male and female subjects [1], [2]. The first dataset was Guerreiro et al. (2013) in which they studied *Trem2* genetic variation based on the genome, exome, and Sanger sequencing results among 1,092 Alzheimer’s disease individuals along with 1,107 normal individuals as controls. They found that based on their meta-analysis results *Trem2* variant rs75932628 (encoding Arg47His) was the most commonly associated variant, rs75932628 was highly significant associated with Alzheimer’s disease (P<0.001) and *Trem2* expression varied among mouse model of Alzheimer’s disease and control mice. In another report recently published report, Jonsson et al. (2013) observed 2,261 Icelanders and found that those with a variant or defect in the *Trem2* gene had almost a three-fold increased risk of Alzheimer’s disease than those without the mutation. These two findings resulted in the discovery of a variant rs75932628, which was found to be associated with a triply increased risk of Alzheimer’s disease. We performed the follow up analysis on genetic *Trem2* variant (R47H) because of its potential implication in neuroinflammation and pathogenesis of Alzheimer’s disease, which may assist to find rapid and extensive exploration of therapeutics with in this domain to potentially treat the disease.

### Protein Structure Modeling and Altered Protein Regions

We used a modeling method because the studied protein structure does not exist in PDB. To ensure the prediction of the three-dimensional structure for the variant Arg⇒His codon at position 47 on chromosome 6, the prediction was determined using I-TASSER [4]. From this algorithm, the conformation with the lowest energy was selected. The I-TASSER server returns the best 5 models according to C-score that is attached to each individual model. The C-score is a confidence rating that I-TASSER uses to estimate the overall quality of the predicted model. The TM score was below 0.3 according to TM-align and makes reference to the similarity between random structure pairs. The C-score is often reported within the range from −5 to 2; larger C-scores reflect a model with a high level of confidence, and vice versa. To select one of the models, the largest cluster had one of the best C-scores. The calculation of the C-score relies on the significance with respect to the threading template alignments and the convergence parameters of all of the structure assembly simulations. Furthermore, we identified such unstructured or disordered regions within the TREM2 protein based on the amino acid sequence. GlobPlot (http://globplot.embl.de) plots the tendency reports within our TREM2 protein for order/globularity and disorder [5].

### Prediction of the Functional Effect and Stability Analysis

For the overwhelming majority of level mutants (single amino acid adjustments or nsSNPs) in humans, the impact on protein function remains unknown. This process provides binary classifications (impact/neutral) accompanied by a more detailed score. Furthermore, we gained insights about the protein’s stability using Schrodinger (BioLuminate). The structure primarily bases the mutant stability predictions on a protein of unknown structure. In the mutated form, the place of the mutated residue is specified, in addition to the wild type and mutant amino acids. Many known disease-associated nsSNPs in proteins with a known three-dimensional protein structure impact structurally important residues and sites that are relevant for function. Disease causing mutations typically occur within the protein (buried) and at hydrogen bonding residues [6]. In protein kinases, comparisons appear to cluster within the functionally important catalytic core [7], followed by residue scanning to fix polar and neutral residues for analysis of the protein stability and solvent accessibility. Additionally, we also compared the predictions of the functional effects that were determined by the Screening for Non-Acceptable Polymorphisms (SNAP) [8]. SNAP scores differ from −100 (strongly predicted as neutral) to 100 (strongly predicted to alter function); the distance is directly related to the binary determination boundary (0), which measures the reliability of the impact [9]. To illustrate, disease-associated mutations could potentially affect protein interactions [7]. Protein function will typically be associated with evolutionarily conserved residues [10]. A damaging signal corresponds to a mutation that is predicted as being stabilizing. Modifications in the folding free energy upon mutation (ΔΔG) support the notion that a mutation causes disease primarily because it damages an important protein. SNAP scores pertain to extra functional effects [11].

### Structural Effect from Single Point Mutations

Structural impact has been assessed according to the findings of undertaking Have yOur Protein Explained (HOPE) [12], which was developed at the Centre for Molecular and Bio-molecular Informatics (CMBI), Department of Bioinformatics, Radboud University. These approaches analyzed the impact of the mutation, and the findings indicated a structural impact. This report also shows equivalence to contacts such as metal, DNA, hydrogen bonds, and ionic interactions and evaluates whether a mutation impacts an essential contact, structural areas together with motifs, domains, and trans-membrane domains. These tools make certain that the foremost dependable method to acquire data and facts is used, i.e., data regarding the “actual protein structure” that provide annotated information in UniProtKB that is utilized by prediction with DAS-servers [13]. Initially, we checked whether the mutation was situated in a field in the protein that included an assigned and corresponding function, with non-structural features such as posttranslational modifications based on a protein sequence in FASTA format. In addition, this approach could effectively note the mutated residue. These analyses provide beneficial options to study that could indirectly have an impact on the structure of the protein. Finally, our report addressed how the protein structure was affected by the impact of the mutation.

### Molecular Dynamics (MD) Simulation

Schrodinger (New York, NY, USA.) and Tripos Sybyl-X (Corporate in St. Louis, Missouri, USA.) were used to evaluate a number of entered data records and molecular systems and to standardize and generate the utilization of common, as well as advanced, simulation strategies in CHARMM. The contact energies were determined using MOE (Molecular Operation Environment, Chemical Computing Group, Canada). The MD simulations were carried out with a 5-fs time method using a constant temperature of 300K along with an invariable pressure of 1 atm below the periodic solvent boundary conditions. The solvated system was neither minimized nor equilibrated. These research programs are used extensively for macromolecular mechanics and dynamics and serve as resourceful evaluation and manipulation tools for the atomic coordinates and the dynamic trajectories. These simulations commonly follow two strategies, specifically energy minimization and molecular dynamics, which, respectively, optimize structure and simulate the natural motion of biological macromolecules. We also performed a conjugate gradient technique for 3D structure optimization; additionally, the deviation between the structures was assessed according to their RMSD values. The program Gromacs was used for forcefield energy minimization and was started with a steepest descent, conjugate gradient, and Limited-reminiscence Broyden Fletcher Goldfarb Shanno (L-BFGS) methods [14]. The free energy simulations were achieved with lesser number of solvent water molecules towards the solute, with complete process of solvent mass by attaining appropriate Solvent Boundary Potential (SSBP). The initial ions configuration was confirmed using brief Monte Carlo (MC) simulations with a basic model, for example, vdW interactions. The Particle Mesh Ewald methodology [15] was used for electrostatics, and a 12 Å cutoff was applied for the vdW interactions. In 51M, ions may be added in the simulation box by specifying ions (KCl) with a focus (C). A knowledge-based potential derived according to our TREM2 protein domain structure was used to check the energy function implicitly and protein alterations for the results of the solvent together with the crystal environment. The solvation free energy was expressed as the nonpolar and electrostatic contributions; however, the nonpolar contribution was once more partitioned into the repulsive and dispersive contributions. The MD simulation techniques were performed by Desmond of D. E. Shaw Research [16] and were used to analyze the trajectories over 10 ns, which were analyzed by calculating the RMSD of TREM2 at 300K. At the initiation of the trajectory (t = 0), the RMSD of TREM2 had a value of 0.6 Å, which indicated that movements occurred during the thermalization and equilibration periods. At 300K, the deviation value increased rapidly during the first 3 ns, and the deviation was maintained until the end of the simulation (t = 10 ns). The conformational structure of the TREM2 Arg47His structure continuously changed with the temperature during the MD simulations at 50 to 70°C until the end of the simulation (10 ns). In addition, a comparison was performed between the structures of the wild type and mutant forms for simulated annealing with parallel tempering. To perform a relevant comparison, simulated annealing simulations were run with a linear cooling schedule with the same temperature determined by Tripos (Sybyl-X). To fix the parameters, the forcefield was used to calculate a time set of 1.00 fs, a constant temperature of 300K, a temperature coupling factor of 100.00 fs, and a simulation interval of length 1.0000.00 fs.

## Results

### Residue Scanning and Loop Mutation, MD Simulation of Native and Mutant Proteins and Solvent Accessibility of Amino Acid Residues

**Figure 1:**
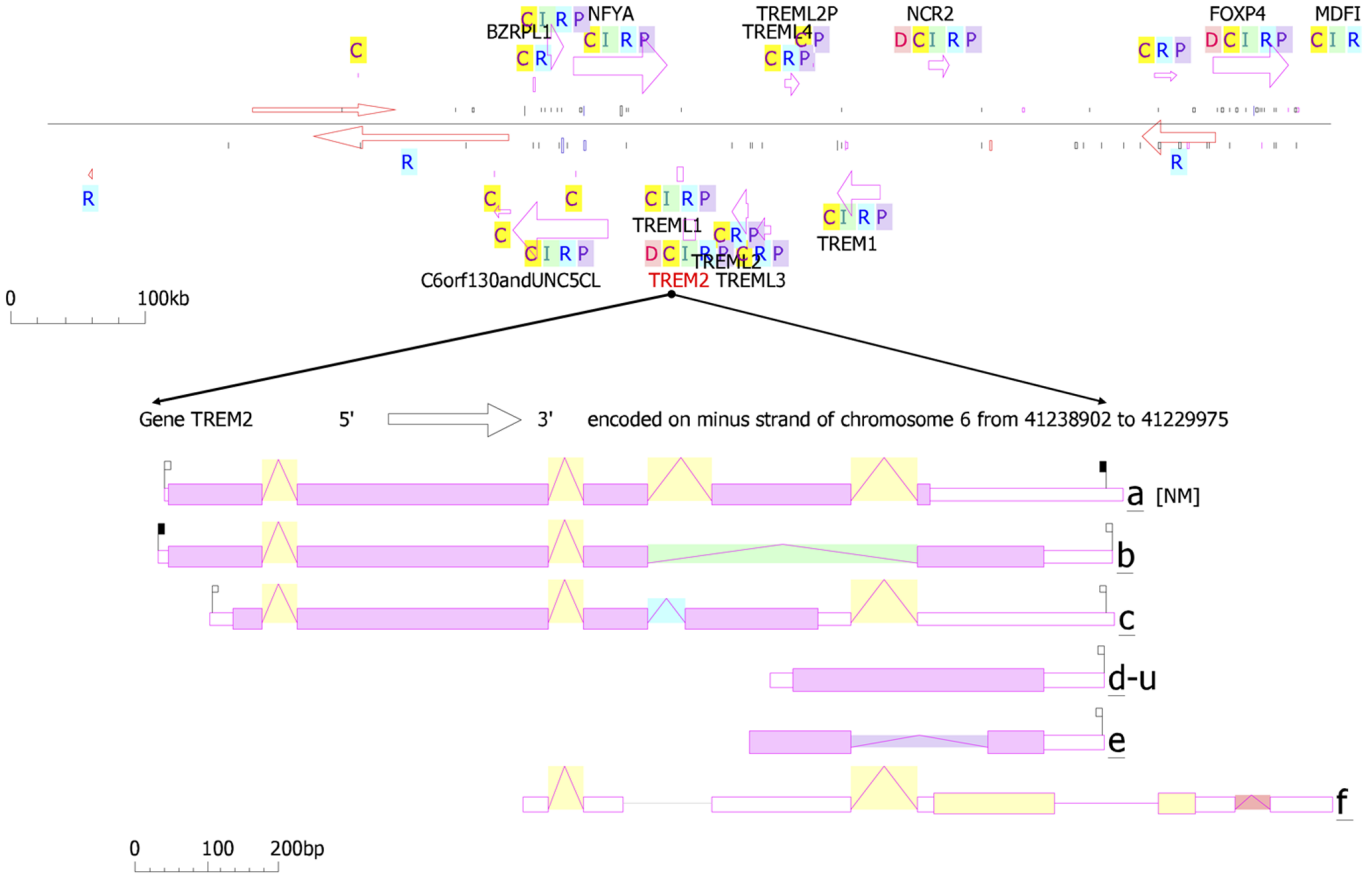
TREM2 Gene annotations with colored pastilles. Alternative mRNAs are shown aligned from 5′ to 3′ on a virtual genome where introns have shrunk to a minimal length. The exon size is proportional to the length; the intron height reflects the number of cDNA clones that support each intron. The genes are summarized according to color as follows: gene (pale blue) known to Entrez, disease (red), conservation (brown), interactions (green), and regulation (dark blue). Each arrow represents a gene and covers the extent of the GenBank/dbEST cDNA sequences that belong specifically to the gene and points in the direction of transcription (top strand is up, bottom down).

**Figure 2:**
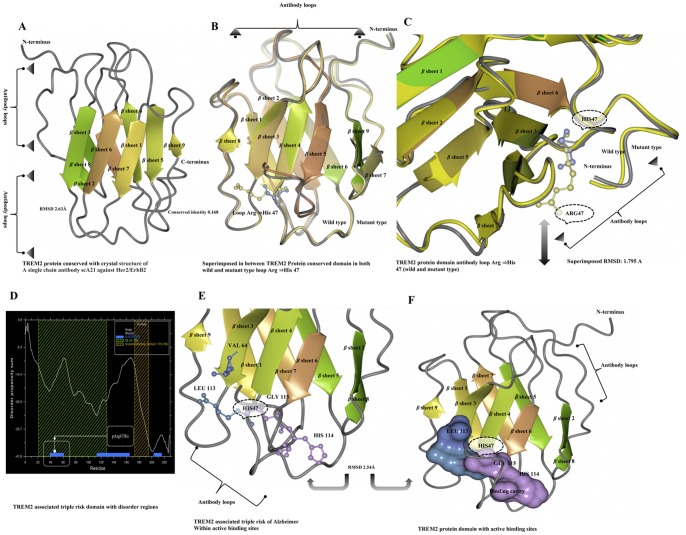
TREM2 protein domain structure modeling and annotations. **A)** TREM2 protein domain conservation, according to the crystal structure of a single chain antibody scA21 against Her2/ErbB2 with a conserved identity of 0.168 and RMSD of 2.63 Å. **B)** Ribbon diagram representation of a composite model superimposed structure between the conserved domain of TREM2 between the wild type and mutant forms of the loop region at *β* sheet4: Arg ⇒His47. **C)** TREM2 protein domain antibody loop in the wild type (yellow) and mutant (violet). **D)** Predicted altered regions of the TREM2 associated triple risk protein domain. **E)** Predicted active binding site of wild type Arg (blue) and mutant-type His (violet) at the loop region of TREM2 associated with a triple risk of Alzheimer’s. **F)** The molecular surfaces of the TREM2 domain model. The molecular surfaces at 2.54 Å RMSD of the active binding sites in wild type (blue) and mutant (violet) forms; the most positive potential is shown in blue, and the most negative is shown in deep violet.

**Figure 3:**
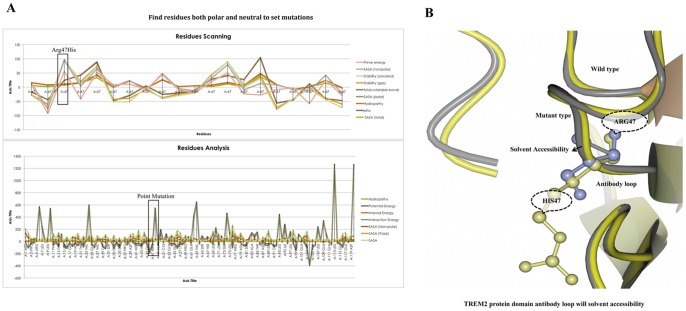
Scanning of residues with functional annotations, prediction of solvent accessibility, and protein stability changes. **A)** Compositions of the amino acids in TREM2 protein residues, fixing only the mutant His47 in comparison to the polar and neutral residues for the solvent with a random representative set of non-homologous TREM2 proteins. On the Y-axis is the percentage of amino acids, and on the X-axis is the amino acids regions compared with the functional annotations in different colors as follows: prime energy, SASA (non-polar), stability (gas), total rotatable bonds, SASA (polar), hydropathy, pKa, the SASA (total) interactions, and the solvent-accessible contact area as a percentage of the residue accessibility at the point mutation. **B)** TREM2 protein domain antibody loop, according to the solvent accessibility compared to the wild type and mutant residue stability. The gray color loop shows the solvent accessibility of the mutant residue (yellow), and the wild type (violet color) residue are shown in violet.

**Figure 4:**
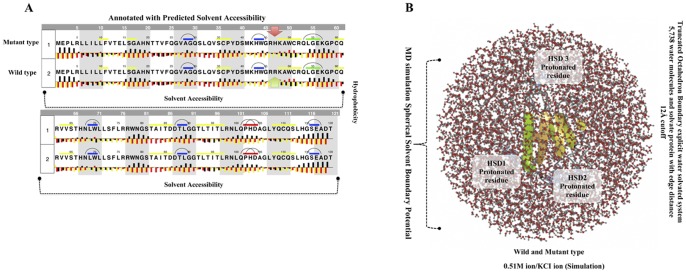
Evaluation of the secondary structures of the TREM2 domain and MD simulation in solvated protein. Values of median pho (black bars) and phi (yellow bars) ASA for each residue in the extended state (**A**) with residues sorted according to decreasing total ASA values. Median hydrophilic solvent accessibility as a function of median hydrophobic solvent accessibility of residues was in most of the loop regions. Residues of wild type Arg47 (R) (green) and mutant His (H) (red) are upstream outside of this classification. The distribution of total a solvent accessibility for wild type and mutant (Arg ⇒His47) residues. The values of the median and mean total solvent accessibility are indicated for each of these residues. **B)** The MD simulations used in the calculations included a water box surrounding the entire protein (middle) and the tertiary structure of the wild type TREM2 protein domain showing triple risk associated mutations, i.e., Arg ⇒His47 (R→H) on *β* sheet4 is shown in green. The visual inspection also allowed us to identify the side chain of a histidine residue involved in the hydrogen bonding with surrounding molecules and, in that case, the δ nitrogen of the histidine (HSD1-3) was a protonated residue.

A point mutation that causes an amino acid alteration can drastically modify the stability of the resulting protein structure; therefore, the modeling of protein structural data will likely be a requisite for the understanding of protein functionality. The SNPs were acquired from a database of SNPs (dbSNP) (http://www.ncbi.nlm.nih.gov/SNP) [17], and general information about SNPs was obtained from both the dbSNP and the Human Genome Variation database (HGVBASE) [www.hgvs.org], which provide information for the translated regions of the gene. When a mutation is located in a gene region that is translated into a protein (in an exon), it must be considered that the mutation is a missense mutation for the protein. The *Trem2* gene has not previously been investigate via *in silico* structural and functional studies using molecular dynamics (MD) simulation. The annotation for the triple risk single nucleotide polymorphism (SNP) within the gene on chromosome 6 was performed (Figure 1). Although there is no available protein structure information in the Protein Data Bank (PDB) database, we resolved the structure predictions from UniProtKB (Q9NZC2: TREM2_Human), which starts from the residues of the 1 to 120 bp region for homology modeling, and the CLCbio Genome Workbench utility (Denmark) determined these predictions. The I-Tasser server was used to predict the protein structure for the selected regions. The most effective five models were used to optimize the C-score for each specific model. These processes were selected from the top 10 templates applied to the threading. The best C score obtained from our models was −0.66. The predicted structure had extremely good similarity with the X-ray diffraction structure of the Anti-Methotrexate CDR1-4 Graft VHH Antibody in Complex with Methotrexate (PDB ID: 3QXV) (Fig. 2*A*). This structure is typically created from four chains, but our sequence aligned with only “Chain D” alignments for the convergence parameters with regard to the structure assembly simulations. Our model was composed directly from the largest cluster, which provided one of the best C-scores when estimating the accuracy of the model; the scores were 0.63±0.13 (TM-score) with 11.5±4.3 Å (RMSD), the number of decoys was 1,926, and the cluster density was 0.0433. The predicted TREM2 associated protein domain structure mutation was within *β* sheet 4 at Arg47His. The mutations for corresponding positions were achieved utilizing CCP4 (QtMG) viewed individually to examine the altered model structures (Fig. 2*B and 2C*). The predicted TREM2 evaluation was determined using a Ramachandran plot with a favored region (98.0%), an allowed area (2.0%), and an outlier region (10.4%). We found greater than 90% of the residues in the most favored regions, which showed that the quality of the predicted structure was comparable to the template [18]. To predict the protein disorder binding regions, we sought to identify segments in disordered regions, which cannot form sufficient favorable intra-chain interactions to fold on their own and are likely to gain stabilizing energy by interacting with other globular proteins. Altered protein regions demonstrate an affinity with consideration to the structural environment, although the individual quantities that are combined are selectively better with respect to the various types of residues. The effect of these alterations was evaluated by comparing the amount of altered amino acids in the short disordered binding sites. The TREM2 protein demonstrated several altered portions, especially in the regions 40–50 amino acids (Fig. 2*D*). Our target residue, *β* sheet 4: Arg47His was located within one such altered region (Fig. 2*D*), and we recognized that these binding sites contained SNP rs961253 within the predicted sites. To validate their regulatory function, the location that the SNP was predicted to have an effect on binding is usually considered to be heterozygous (Fig. 2*E and 2F*). The residues were scanned, and loop mutation analysis was performed for TREM2. The consensus method was used on an additional critical residue with a hot residue to increase the thermodynamic stability of TREM2, and this technique was simple in comparison to previously described strategies [19]. Arg47 in TREM2 and another thermo stable site (His47) were targeted as critical residues and were substituted using Schrodinger (BioLuminate). The energy minimization of TREM2 protein are before the mutation was performed at −471 971.985 kJ/mol, which decreased to −507 569.663 kJ/mol after mutation, as calculated by using BioLuminate. The energy minimization was based on the set of selected polar and non-neutral residues for these screenings. We fixed the refinement cut of 0.00 Å with an implicit solvent minimization. The result of decreasing the minimized energy value of the *β* sheet 4: Arg47His showed that the substitution of Arg47 to His may contribute to improved compactness and may increase protein folding (Fig. 3*A and 3B*). Further loop mutations were based on the implicit solvent according to MD simulations. During these simulations, proteins continuously fold and unfold and provide considerable insight into this process [20]. Compared to the results at a temperature of 300K, the deviation of the TREM2 protein increased from 2.0 Å to 10 ns. In contrast, the deviation of mutation Arg47His was maintained at 1.2 Å until the end of the simulation (t = 10 ns), which showed that Arg47His had reached its folded state. The small peak at 1.7 ns indicated that Arg57His stabilized the structure. Our results showed that the Arg47His structure was stable and could maintain its conformation at 300K, at a pressure bar of 1.01325 with a surface tension 4,000.0 Å, in a total simulation time (ns) of 1.2/elapsed 0.0, and a recording interval (ps) energy of 1.2. However, there were subtle changes that were observed between wild type and mutant TREM2 (*β* sheet 4: Arg47His), as revealed by the superimposed structures with a RMSD value of 1.795 Å (Fig. 2*C*), which were determined using MOE. As shown in Figure 3*B*, Arg47 of the TREM2 protein showed a higher solvent-accessible surface area than His47 of the mutant inside the protein structure. The removal of Arg47 (less hydrophobic properties) inside of the protein structure followed by the addition of a His residue was able to increase the compactness and remove the bad cavities in the protein structure. The annotated predicted solvent accessibility is shown in Figure 4*A*. In a further analysis of the pre-calculated packing density, Arg47His was found to increase the density and lessen the internal cavities compared to the wild type form. Thus, substitution of an altered residue (Arg47) with His enhanced the packing and compactness of the mutant structure and decreased the internal cavities in the protein structure. The consequences of the mutation were studied using a classical molecular dynamics approach, whereby each of the native and mutated structures were assessed via long simulations in explicit solvent, and the variations in the dynamics and stability were also investigated according to the TREM2 associated protein domain structure and computations of free energy. The solvate was utilized to accumulate successful aqueous solvent surroundings near to the TREM2 associated protein domain structure with water. The solvate identified the dimension of the system with octahedral designs of water boxes adjusting to fully solvate the molecule with an edge distance of 10.0 Å (Fig. 4*B*).

### MD Simulations of *TREM2* Protein Conformational Changes of RMSD and RMSF

**Figure 5:**
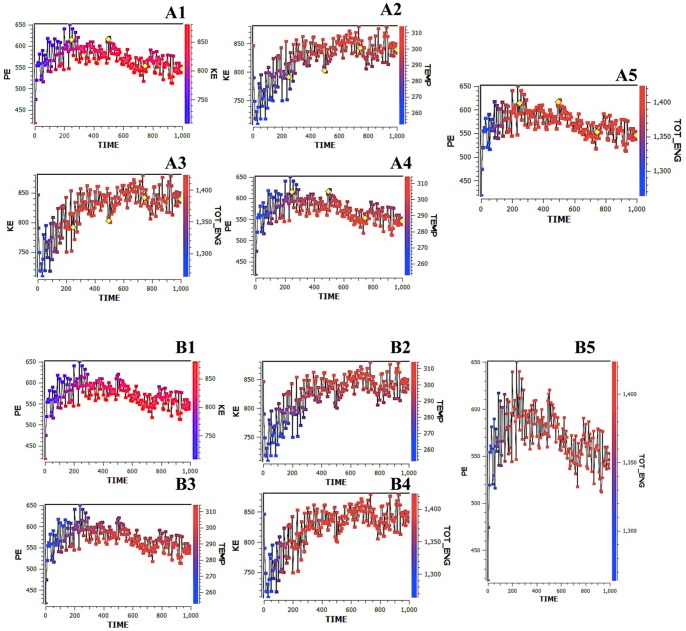
Simulated annealing of TREM2 Protein by multiple-time-step molecular dynamics. Energy refinement calculations of restrained minimizations and dynamics were carried out on the best distance geometry structures using the SYBYL-X program executing the Kollman all-atom force field. The structures of TREM2 were also obtained using simulated annealing on the protein with the distance constraints from the obtained data. Simulated annealing was performed with the Kollman All Atom force field and Kollman charges within Sybyl-X. The molecule was heated to 300 K for 1.0000.00 fs followed by cooling to 200 K during 100.00 fs. These calculation cycles were performed on a super-computing cluster with silicon graphics. The distance residues of geometry simulated parallel annealing and dynamics, as shown as (A1–A5). **A1**: X-axes of the potential energy (PE) and kinetic energy (KE). **A2**: X-axis of KE and temperature (T). **A3**: X-axis of KE and total energy (TOT-ENG). **A4**: X-axis of PE and (T) temperature. **5**: X-axis of PE and TOT-ENG. The highlights are of the distance residues (yellow) and are closed with the Arg47His mutation. Furthermore, the starting structure as a function of the simulation time is given. TREM2 protein domain calculations were performed for the backbone atoms of the respective structure with the dynamics simulation method, as shown in the figure (**B1–B5**).

MD simulation techniques, as determined by Desmond, were used to analyze the dynamic behavior of TREM2 protein. Trajectories over 10 ns were analyzed by calculating the RMSD of TREM2 at 300K. At the beginning of the trajectory (t = 0), the RMSD of TREM2 had a value of 0.6 Å, which indicated that the movements occurred during the thermalization and equilibration periods. At 300K, the deviation value increased rapidly during the first 3 ns, and the deviation was maintained until the end of the simulation (t = 10 ns). At the same time, Arg47His showed a rapid increase in the deviation value (1.6 Å) until 8 ns and then decreased until the end of the simulation. The Arg47His structure was not stable and did not reach the folded state until the end of the simulation at 50°C. However, it is possible that, at simulation times that were longer than the 10 ns initially performed, the Arg47His structure may be stable and reach the folded state. MD simulations over a long time scale are important for increasing our understanding of the dynamic aspects of protein structure. During longer time simulations, proteins continuously fold and unfold. In contrast to the temperature at 300K, the deviation of the TREM2 increased until 2.0 Å at 10 ns. In contrast, the deviation of the mutated Arg47His was maintained at 1.3 Å until the end of simulation (t = 10 ns), which showed that Arg47His had reached its folded state. The result at 1.8 ns explained that Arg57His was able to stabilize the structure. Our results indicated that the Arg47His structure was stable and could maintain its conformation at 300K (60°C); at the same time, TREM2 was stable at 300K/50°C. It was shown that the substitution of Arg47 with His increased the thermal stability of the protein structure in the mutant with regard to the longer stay in the folded state at a higher temperature. The folded state of the protein was compact and rigid but became flexible because of vibrational and conformational entropy. However, for the unfolded state [21], [22], the increase in the kinetic energy of the polymer molecules increased the conformational changes and the elasticity. Furthermore, the root mean square fluctuation (RMSF) of the Arg47His residue of TREM2 was used to calculate the trajectory of the overall flexibility of the system at a temperature of 300K. A slight fluctuation was observed in the TREM2 protein sequence at 1–200, which was located at the flexible region of TREM2. The flexible region comprises a *β* sheet and a loop that is located on the surface of the TREM2 protein domain structure. The fluctuation of the flexible region of TREM2 then increased the flexibility at a higher temperature (300K/60°C). In addition, we found that the flexible region was a thermally sensitive region. The critical point mutation increased the protein folding and compactness as a result of less flexibility in the flexible region. The trajectories from the MD simulations performed over 10 ns for the TREM2 Arg47His structure was sampled at two temperatures (50 and 60) to analyze the differences in the structural stabilities. The N-terminal of the TREM2 protein was evaluated in the structures before and after the simulations (for 10 ns) and at different temperatures. A large fluctuation in the flexible region would trigger an unfolding process and, consequently, would denature the protein [23]. The conformation of the TREM2 Arg47His structure continuously changed during the MD simulations at 50, 60, and 70°C until the end of the simulation (10 ns). Thus, in our study, the conformational changes did not correlate with the thermal stability but, instead, indicated that greater compactness and rigidity were the major factors determining the protein thermal stability. Arg47His had a compact and rigid protein structure because of its ability to remain stable and rigid at a high temperature (60°C), and further confirmation was obtained using parallel-simulated annealing molecular dynamics. Furthermore, we compared the structures of wild type and mutant simulated annealing to simulated annealing with parallel tempering. To perform a relevant comparison, simulated annealing simulations were run with a linear cooling schedule at the same temperature determined by Tripos (Sybyl-X). In a forcefield calculated with a time of 1.00 fs, a simulation interval length of 1.0000.00 fs, a constant temperature of 300K, and a temperature coupling factor of 100.00 fs, the potential energy was calculated as 978.464 kcal/mol, the kinetic energy was 1,131.403 kcal/mol, and the final total energy was 1,823.599 kcal/mol. These temperatures could efficiently overcome large potential barriers and, at low temperatures, selectively gain access to low-energy structures of the TREM2 protein. One advantage of parallel tempering simulations over simulated annealing is that an exchange between low and high temperatures is possible throughout the entire simulation. Efficient exchanges between replicas can take place only if there is some overlap between the potential energy of neighboring replicas over the course of a simulation. It has been shown that the number of replicas needed in a parallel tempering simulation scales approximately with the square root of the number of particles. Furthermore, simulations with a large number of replicas require increased simulation time for the replicas to ‘diffuse’ over the range of temperatures and to effectively cross barriers. An example of a parallel tempering simulated annealing molecular dynamics is given in Fig. 5*A* *1–A5* and 5*B1–B5*.

### Disease-Causing Mutation Strongly Predicted to Alter Protein Structure and Function

The protein structure of mutant Arg47His was located within a domain that was annotated in UniProt as an “Ig-like V-type” domain. The mutation introduces an amino acid that has different properties, which can disturb the domain and abolish its function. The wild type amino acid was highly conserved, however some other residue types have also been observed at this location (position 47). Neither the mutant residue nor another residue type with similar properties was observed in other homologous sequences. Based on the conservation scores, this mutation was deemed likely damaging to the TREM2 protein domain. The mutant residue was located near a highly conserved area and was part of an interpro domain in both “Immunoglobulin V-set” (IPR013106) and “Immunoglobulin-like fold” (IPR013783) domains. Immunoglobulin (-like) domains are involved in a wide variety of functions that usually require interaction of the intact domain with another protein/molecule, and a mutation in such a domain could disturb this interaction. The mutated residue was located on the surface of a domain with an unknown function. The residue was not found to be in contact with other domains for which the function was known within the TREM2 protein domain. The wild type residue was conserved, but other residue types were also observed. Neither the mutant residue nor another residue with similar properties was found at this position. This mutation will, therefore, likely be damaging to the protein. The charge of the wild-type residue was lost as a result of this mutation, and this change can cause a loss of interactions with other molecules. This change may also cause a deficiency in external interactions. However, contact with other molecules or a domain was still possible and may also be affected by this mutation.

### Effects of Single Amino Acid Substitutions on Protein Function and Mutation Effects

The Alzheimer’s disease tripled risk variant *β* sheet 4: Arg47His revealed changes in single amino acids among the many amino acids in the protein sequence. The SNP collection indicated that there is also a large amount of genetic variation at this location. If the method showed a SNP as “neutral”, then the resulting point-mutated protein will not be functionally distinct from the wild-type form. However, if it is “non-neutral”, then the mutant and wild types will differ functionally, and this will have an impact on the fitness of a specific phenotype; a non-neutral mutation could cause a disease. The targeted rs75932628-T SNP in this study was found to be “non-neutral” based on the output score. The results indicated a RI > = 0 binary score, which could be translated into a binary prediction effect and reliability index (RI) measurement, which resulted in an expected value of 80% with a highly significant risk of Alzheimer’s disease.

### Protein Structural Loop Stability from the Arg47His Substitution and Energy Contacts

**Figure 6:**
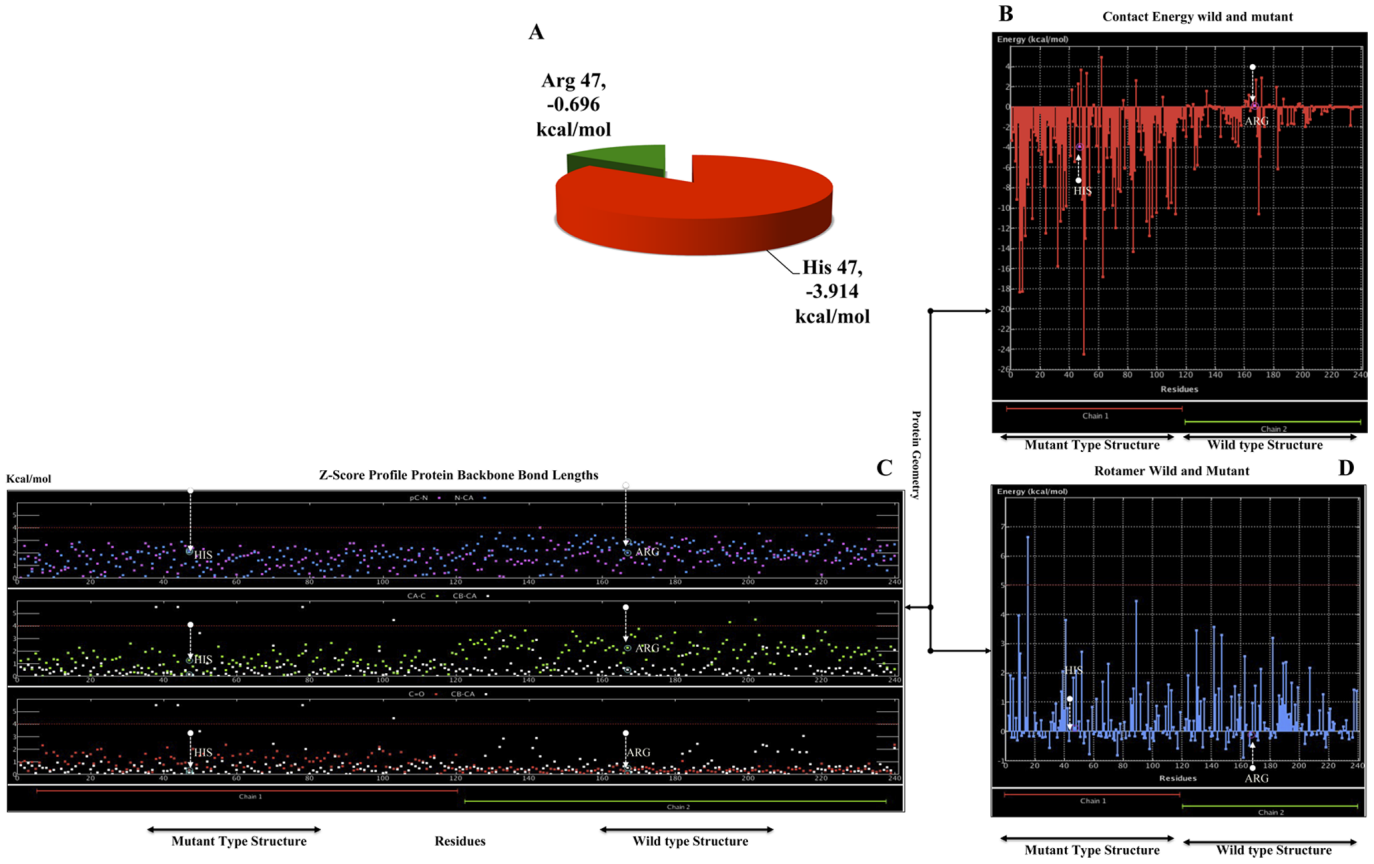
Contact energy and protein geometry of the TREM2 domain. **A)** Pie chart showing the energy significance for wild-type and mutant residues of Arg⇒His47, i.e., wild-type Arg47 (green) −0.696 kcal/mol and His (red) mutant type −3.914 kcal/mol. **B)** The constructed TREM2 domains for wild-type and mutant positions of the amino acid residues are shown on the x-axis, while the contact energies are shown on the y-axis. The chain 1 (red) mutant type structure of residue His47 energy was shown on the y-axis is indicated by the arrow shown in the downstream region (white), but chain 2 (green) of wild type residue His47 energy is shown as the x-axis and is indicated by the arrow shown in the upstream region (white). The trends in the variation of the contact energy in most of the parts of the TREM2 domain were in good agreement with that of the X-ray structure of TREM2. **C)** The rotamer wild type and mutant structure residue positions are shown on the x-axis, although the contact energies are shown on the y-axis. The chain 1 (red) mutant type structure of amino acid residue His47 energy was shown as the y-axis indicated by the arrow shown in the upstream region (white), but chain 2 (green) of wild-type residue His47 energy was shown as the x-axis indicated by the arrow shown in the downstream region (white). **D)** The protein backbone of both mutants was shown in red (chain-1), and the wild type from is shown in green, with a bond length that is based on the Z-score profile of the protein geometry. The distribution of three portions is given: 1) [PC-N (pink), N-CA (blue), 2) CA-C (green), CB-CA (white), and 3) C = O (red), CB-CA (white).

The effects of a critical point mutation on thermodynamic stability were used to study BioLuminate and MOE. The TREM2 Arg47His mutants were analyzed using MD simulations at various temperatures, starting at 300.0K at a time step of 1.00 fs. The coupling factor temperature increased the pressure bar at 100.00 fs. At the same time, the stability increased in the loop from 300K. The thermal transition curve obtained with temperature dependence at 1.00 fs also indicated that the mutant form increased the thermostability. Moreover, as temperature is directly related to protein stability, it was assumed that the substitution of a less hydrophobic residue (Arg47) for a more hydrophobic residue (His) increased the internal hydrophobicity of Arg47His to maintain the structural stability at a high temperature. Choi et al. [24] reported that the wild type (Arg47) loop structure was comparable to the mutated (polar and neutral) residue. The MOE of the protein geometry suggests that altering amino acid 47 from Arg to His yields excessive folding-free energy (ΔΔG = −3.914 kcal/mol) (Fig. 6*A*), which indicates a destabilizing impact on the TREM2 protein domain structure when compared to both the wild-type and mutant forms (Fig. 6*B*; 6*C* and 6*D*). Moreover, the mutant tertiary structure generated by the I-Tasser model predicted that an alteration from Arg to His at amino acid position 47 would lead to significant perturbations in protein folding, especially in the region with a correlation coefficient of 0.8 between the predicted and measured stability changes (using cross-validation and after the exclusion of 10% outliers). The best value obtained was 0.67. Our findings predicted stability modifications resulting from the mutation in the TREM2 protein domain, as may be observed directly from the relatively low correlation coefficients obtained using different approaches for the Arg47His mutant. A considerably larger than average concentration of structural weaknesses was detected, thereby quantifying how these sites appear to have been optimized for function instead of for stability.

## Discussion

This study was conducted based on two recently published reports using comprehensive series of genetics methods, involving direct sequencing of DNA from Alzheimer’s patients and controls along with genome-wide association studies with imputation as well as assays of gene expression across regions of the human brain and in mouse models. Jonsson et al. (2013) discover that the Arg47His variant of TREM2 was highly associated elevated risk of Alzheimer’s disease among a homogenous group of Icelanders as well as in other, more heterogeneous, individuals. Likewise, among individuals from European and North American ancestry, Guerreiro et al. (2013) established a very high risk for Alzheimer’s disease linked with the Arg47His. These studies implicated a strong association between the Arg47His variant and Alzheimer’s disease and found that the variant resulted in poorer cognitive skills old age individuals without Alzheimer. The identification of TREM2 variant is essential and will play a significant role as it poses a high risk for Alzheimer’s disease and due to the fact that the gene’s normal biological function resulted in reduced immune function which probably adding to disease onset. This discovery has two main consequences. First, this observation provides a better understanding of the immune system’s involvement in Alzheimer’s disease, which involves the gene for complement receptor 1 (CR1). The gene was a previously implicated in a study by Inserm-Lille2-IPL UMR744 [2], British, American, and French researchers have now shown that, on this same region of chromosome 6, mutations in the TREM2 gene are associated with a five-fold increased risk of developing late-onset Alzheimer’s disease. Complete sequencing was performed on 281 individuals with Alzheimer’s disease and 504 controls, and the analysis of the TREM2 gene showed excessive TREM2 mutations in those with the disease compared to control subjects. The characterization of one of these TREM2 mutations in very large sample of patients with Alzheimer’s disease has allowed researchers to measure precisely the importance of this association between TREM2 mutations and disease. Finally, these authors reported that a replication study was performed in another independent series of 1,994 cases and 4,602 controls, which confirmed this strong association (OR = 4.97 CI 95% [2.42–10.21], P<6.10-6). These results were also confirmed in the same edition of *The New England Journal of Medicine* by an Icelandic team who found that this gene serves as a risk factor for Alzheimer’s disease in the Finnish population as well as other European populations [2]. This study was the first to report that the *Trem2* gene is associated with a triply increased risk of Alzheimer’s disease. The high throughput MD simulation method predicted that the Arg47His allele has an impact on both the functional effect and the structural affect of this mutation. Point mutations as a results of single amino acid modifications may significantly alter the stability of protein structure; therefore, the predicting protein’s structural characteristics is important for a comprehensive understanding of the functionality of a given protein. Our results concerning the translated regions of the gene suggest that this mutation is located in a region where the gene is translated to a protein (in an exon) and that it could therefore result in a missense mutation. Although there is no available structural data in the PDB database, we made predictions about TREM2 protein structure based on its amino acid sequence using I-TASSER. The amino acid placement of this mutation was within *β* sheet 4 at Arg47His; The TREM2 form associated with the triple risk nucleotide allele was “T”; otherwise, it was wild type. Furthermore, the consequence of the Arg47His mutation was previously studied by Schrodinger (BioLuminate) using classical molecular dynamics strategies for analyzing both the native and mutated structure with long simulations in explicit solvent along with investigations into protein dynamics and stability. These variations were investigated within the TREM2 protein domain structure with the purpose of computing the free energy. The energy minimization studies were performed with the native TREM2 protein domain as well as the mutant type structure. The prediction of disordered regions is also important for the functional annotation of proteins. In the sense of the classical ‘lock-and-key’ theory by Emil Fischer in 1894, it is difficult to imagine that natively disordered regions have biological meaning. However, disordered regions are reportedly involved in many biological processes, such as regulation, signalling, and cell cycle control [25]. We targeted the regions based on the studies of Guerreiro et al. (2013), where they used the genome sequences of 2,261 Icelanders, and recognized the sequence variants that likely affected the protein function. We identified our triple risk associated SNP rs75932628-T Arg47His, which was situated in the altered region of the TREM2 protein domain structure. Human TREM-2 cDNA encodes 230 amino acids, including an 18 amino acids signal sequence, a 156-extracellular domain (ECD) with one V-type Ig-like domain, a 21 amino acid transmembrane (TM) domain, and a 35 amino acid cytoplasmic tail [1]. The 230-amino acid TREM2 polypeptide belongs to the immunoglobulin superfamily (Ig-SF) and is predicted to comprise a 13 amino acid signal peptide followed by a 154 amino acid extracellular domain encoded by exons 2 and 3, with two cysteines that are potentially involved in generating an intra-chain disulfide bridge of the Ig-SF V-type fold. In addition, the 33 amino acid transmembrane domain is followed by a 30 amino acid cytoplasmic domain [26]. The functional and structural studies of the protein structure of mutated Arg47His identified a domain that was annotated in UniProtKB as a “Ig-like V-type” domain. This residue was also part of an interpro domain in both “Immunoglobulin V-set” (IPR013106) and “Immunoglobulin-like fold” (IPR013783) domains. Immunoglobulin (-like) domains are involved in a wide variety of functions that usually require an interaction of the intact domain with another protein/molecule. The residue in question was not found to be in contact with other domains with a known function within the TREM2 protein domain. However, contact with other molecules or a domain remains a possibility and may be affected by this mutation. This paper is the first to examine the specific SNP rs75932628-T, for which we report that the thermodynamic protein stability changed as a result of single site mutations. We used a linear mixture of statistical potentials whose coefficients were determined by the solvent accessibility of the mutated residues and adjustments caused by the Lys25Arg mutation, which had a destabilizing impact on the TREM2 associated protein domain structure. However, point mutations that cause amino acid alterations can drastically modify the stability associated with protein structure, and the modeling of protein structural information is needed for a complete understanding of protein functionality. The antibody loop structures are of the utmost importance, as only a very specific combination of antibody loops will bind to and neutralize a given target. With billions of different possible loop arrangements and sequences, it is seemingly impossible to predict which antibody loops will bind to a specific target molecule. Our report addresses the effect of the critical point loop mutation toward thermodynamic stability. The TREM2 Arg47His position was analyzed by performing MD simulations at various temperatures, starting from 300.0K at a time of 1.00 fs. The coupling factor temperature increased the pressure bar of TREM2 Arg47His at 100.00 fs, and the result showed that Arg47His increased the stability in the loop from 300K. We found that the structures of the mutants were less stable than the wild type forms, according to the RMSD, RMSF and DSSP series. Specifically, unwinding of the secondary structures and distortion of the loops below the helices were observed. These structures may prove to be very useful for novel drug targets on the relevant protein structure, and the models built on this work could therefore be applicable for predicting the impact of SNP rs75932628-T on both protein structure and the function. This approach could be helpful for future research and could be combined with the findings of currently ongoing pharmacogenomics studies to provide a useful model for the further analysis of Alzheimer’s disease risk.

## Conclusions

The annotations of diseases resulting from SNP variants are studied so that they may be used as proxies for the functional impact of disease. These can be identified very effectively using methods that predict the effect of the mutations on protein function. In addition, a long-term follow-up may possibly be significant for judging the survival implications that are associated with these risk alleles. Thus, existing prediction methods can be used to select a group of suspect SNPs relevant for Alzheimer’s disease. This study provides an understanding of the role of genetic mutations in Alzheimer’s disease and will aid in the development of drugs that stimulate an opposite effect on the immune system function to reduce the risk of Alzheimer’s disease.
